# Degradation and Mineralization of Benzohydroxamic Acid by Synthesized Mesoporous La/TiO_2_

**DOI:** 10.3390/ijerph13100997

**Published:** 2016-10-10

**Authors:** Xianping Luo, Junyu Wang, Chunying Wang, Sipin Zhu, Zhihui Li, Xuekun Tang, Min Wu

**Affiliations:** 1 Faculty of Resource and Environmental Engineering, Jiangxi University of Science and Technology, Ganzhou 341000, China; Wangjunyu1025@163.com (J.W.); cywang@jxust.edu.cn (C.W.); beyond_life@163.com (S.Z.); 13177767724@163.com (Z.L.); 2 Post-Doctoral Scientific Research Workstation of Western Mining Co. Ltd., Xining 810001, China; wumin5836@163.com; 3 Jiangxi Key Laboratory of Mining & Metallurgy Environmental Pollution Control, Ganzhou 341000, China; 4 School of Minerals Processing & Bioengineering, Central South University, Changsha 410083, China; 13607974014@126.com

**Keywords:** rare earth doped, La/TiO_2_, characterization, benzohydroxamic acid, photocatalytic degradation, mineralization

## Abstract

Rare earth element La-doped TiO_2_ (La/TiO_2_) was synthesized by the sol-gel method. Benzohydroxamic acid was used as the objective pollutant to investigate the photocatalytic activity of La/TiO_2_. The physicochemical properties of the prepared materials were characterized by X-ray diffraction, X-ray photoelectron spectroscopy, UV-vis diffuse reflectance spectroscopy, specific surface area and porosity, scanning electron microscopy and transmission electron microscopy. As a result, the doping of La could inhibit the crystal growth of TiO_2_, increase its specific surface area and expand its response to visible light, thus improving its photocatalytic activity. La/TiO_2_ with the doping ratio of 0.75% calcined at 500 °C, showing the highest photocatalytic activity to degrade benzohydroxamic acid under the irradiation of 300 W mercury lamp. About 94.1% of benzohydroxamic acid with the original concentration at 30 mg·L^−1^ was removed after 120 min in a solution of pH 4.4 with an La/TiO_2_ amount of 0.5 g·L^−1^. Furthermore, 88.5% of the total organic carbon was eliminated after 120 min irradiation. In addition, after four recycling runs, La/TiO_2_ still kept high photocatalytic activity on the photodegradation of benzohydroxamic acid. The interfacial charge transfer processes were also hypothesized.

## 1. Introduction

In the Gannan area of China, ion-type rare earth ore is widely distributed. Benzohydroxamic acid is a high-effect chelating collector of oxidized ore [1], which is widely used in the flotation of lead oxide ore, copper oxide and rare earth ores [2,3,4]. It belongs to the aromatic alkyl hydroxamic acid, due to the existence of the π-π conjugated effect, which enhances the stability of the chelate compound by increasing the electron cloud density of atomic oxygen. Benzohydroxamic acid has physiological toxicity due to its benzene ring structure, which increases the COD (Chemical oxygen demand) content of mineral processing wastewater [5]. At the same time, N element content is also increased, which would cause the eutrophication when accumulated in the water. Besides, benzohydroxamic acid is difficult to decompose, and it would remain for a few years or an even longer time once released into the water environment. Eventually, it would bring harm to the water environment and would need to be removed.

Photocatalytic technology has a great application potential in sewage treatments [6]. Among the various semiconductor photocatalyst materials, TiO_2_ has been the most promising and widely used photocatalyst due to its unique optical property, strong oxidizing power, nontoxicity, low cost, and chemical stability [7]. However, the wide technological use of TiO_2_ is impaired by its wide band-gap (3.2 eV), which only uses about 5% of the solar light [8,9], and the recombination of photogenerated electron-hole pairs is easy to happen. These drawbacks largely restrict its photocatalytic efficiency [10,11]. Thus, the improvement of photo-catalytic efficiency is still a major challenge in the photocatalysis research field. Doping with ions in TiO_2_ lattice has been proven to be an efficient route to enhance photocatalytic activity [12]. The rare earth element has the special 4f electronic structure, which is able to produce a multi-electron configuration. Its oxide has many characteristics, such as crystal form, strong adsorption selectivity, electronic conductivity and thermal stability [13,14]. Some research has shown that the doping of rare earth ions could effectively improve the photocatalytic activity of TiO_2_ [15,16,17]. La-doped anatase TiO_2_ (101) surface tended to engender oxygen vacancies. The photoelectric conversion efficiency of dye-sensitized solar cells fabricated from 1 mol% La-doped TiO_2_ reached 6.72%, which improved efficiency by 13.5% compared with that of the cells fabricated from pure TiO_2_ [18]. Grujic-Brojcin et al. reported that La-doped TiO_2_ had shown a higher rate of degradation than pure TiO_2_, with a maximum rate for 0.65 mol% La loading [19]. Li et al. studied the catalytic degradation of 4-chloroophenol with La/TiO_2_. The results showed that the 10 wt% La/TiO_2_ has the highest percentage of 4-CP degradation (99.0%) [20]. The enhanced photocatalytic activity of La-doped TiO_2_ might be mainly due to the smaller particle size, larger specific surface area and total pore volume, as well as higher pore structure complexity.

Therefore, numerous studies have been focused on the photocatalytic activities of rare earth doped TiO_2_ in recent years [21,22,23], but there are few studies in the treatment of flotation reagents. With the development of mining, mineral processing waste water has become the main source of pollution in the areas of the mine and its surroundings, especially the residual organic flotation reagent, which has high toxicity and high pollution. As far as we know, there is no similar report on the photodegradation of benzohydroxamic acid by the photocatlaytic oxidation technique.

In this study, La doped TiO_2_ nanoparticles were prepared by the sol-gel method, and their intrinsic characteristics were analyzed by using X-ray diffractometer (XRD), X-ray photoelectron spectroscopy (XPS), UV-vis diffuse reflectance spectroscopy (DRS), Brunauer-Emmett-Teller (BET), scanning electron microscopy (SEM) and Transmission electron microscopy (TEM). The photocatalytic activity of La-doped TiO_2_ samples were evaluated by the degradation rate of benzohydroxamic acid. At the same time, the effects of doping ratio of La, calcination temperature, dosage of La/TiO_2_, pH value, light intensity and other factors on the photocatalytic activity of La/TiO_2_ were investigated.

## 2. Materials and Methods

### 2.1. Materials

The reagents used in this study were analytical grade: lanthanum nitrate (La(NO_3_)_3_·6H_2_O) and tetrabutyl titanate (Ti(OC_4_H_9_)_4_) were purchased from National Medicine Group Chemical Reagent Co. Ltd., Shanghai, China. Anhydrous ethanol (CH_3_CH_2_OH) and acetic acid (CH_3_COOH) were purchased from Tianjin Damao Chemical Reagent Factory, Tianjin, China. Benzohydroxamic acid (C_7_H_7_NO_2_) was purchased from Shanghai EKEAR Bio@Tech Co. Ltd., Shanghai, China (the structure was listed as Figure S1 in the Supplementary Materials). All the experimental solutions were prepared with deionized water.

### 2.2. Preparation and Characterization

In accordance with previous literatures and studies [24,25,26], the La/TiO_2_ catalyst was synthesized by the sol-gel process with the following procedure. A mixture solution of 10 mL tetrabutyl titanate dissolved in 15 mL anhydrous ethanol, with stirring for 30 min, was noted as solution A. Another solution containing 10 mL ethanol, 2 mL acetic acid, 2 mL deionized water, and metal salts (La(NO_3_)_3_·6H_2_O) in the required stoichiometry was noted as solution B. Solution B was added dropwise to solution A under acute agitation to form a transparent, homogeneous sol. The wet gel of La/TiO_2_ was obtained by continuous stirring. After ageing at room temperature for 2 days and then drying at 90 °C, the xerogel was formed. The xerogel was crushed to get fine powder and further calcined in a furnace under air atmospheres. Heating rates were 5 °C·min^−1^ for all samples with 6 h holding time at different temperatures (400 °C, 450 °C, 500 °C) and a cooling rate of 10 °C·min^−1^ to achieve the La/TiO_2_ nano-photocatalyst. The rare earth La-doping ratio was set to 0%, 0.25%, 0.50%, 0.75% and 1% according to the mass fraction of La to TiO_2_.

La/TiO_2_ was characterized with an X-ray diffractometer (XRD) (RigakU Miniflex, Tokyo, Japan) by using monochromatized Cu Kα radiation (*λ* = 0.15418 nm) under 50 kV and 80 mA with the 2*θ* altering from 10° to 80°. The X-ray photoelectron spectroscopy (XPS) analyses of the samples were performed on a ESCALAB 250XI Thermo type multi-function imaging electronic spectrometer (Thermo Scientific, Surrey, UK), monochromatic Al Kα (*hv* = 1486.6eV), 150 W power, 500 μm beam spot. UV-vis diffuse reflectance spectra were achieved by using a UV-vis spectrophotometer (UV-2550, Shimadzu Co. Ltd., Kyoto, Japan), the absorption spectra were referenced to BaSO_4_, and the scanning range was 200–700 nm. The specific surface area and average pore size of La/TiO_2_ were measured by a low temperature N_2_ physical adsorption apparatus (Micromeritics ASAP 2460, Micromeritics Instrument Corp., Norcross, GA, USA). Scanning electron microscopy (SEM) images were obtained by using a TES-CAN VEGA TS-5130SB (Tescan Company, Brno, Czech Republic), the light source of electron-beam was tungsten lamp, and the voltage was 10–30 kV. The morphology of the catalyst was analyzed by a G2-20 Tecnai transmission electron microscope (FEI, Hillsboro, TX, USA). The total organic carbon (TOC) was detected by the total organic carbon analyzer (Vario TOC, German Elementar Company, Hanau, Germany). The FTIR spectra of the composite samples (as KBr pellets) were recorded in transmittance mode using a Nicolet iS5 type Fourier transform infrared spectrometer (Nicolet Instrument Corporation, Madison, WI, USA). The scanning range was 400–4000 cm^−1^.

### 2.3. Photocatalytic Degradation of Benzohydroxamic Acid

The photocatalytic degradation reaction was performed in the light-chemical reaction apparatus (Xujiang Electro-mechanical Plant, Nanjing, China). At first, the suspension was magnetically stirred for 30 min in the dark to ensure the adsorption-desorption equilibrium of benzohydroxamic acid on the catalysts. Then, the irradiation was provided by lamps of different intensity and different ranges of radiation (100 W mercury lamp, 300 W mercury lamp, and 500 W xenon lamp). Approximately 5 mL of reaction solution was taken at given time intervals and centrifuged. At last, UV-vis spectrophotometry was used to measure the absorbance of benzohydroxamic acid under wavelength of 229 nm. The removal efficiency (*R*) was calculated by Equation (1):

(1) $$R(\%)=\frac{C_{0}-C_{t}}{C_{0}}\times 100\%$$

where *C*_0_ is the initial concentration of benzohydroxamic acid, in mg·L^−1^, and *C_t_* is the concentration at reaction time *t* (min), in mg·L^−1^. All the experiments were performed twice at least to control the errors of the experiments.

## 3. Results and Discussion

### 3.1. Characterization

#### 3.1.1. XRD

**Figure 1:**
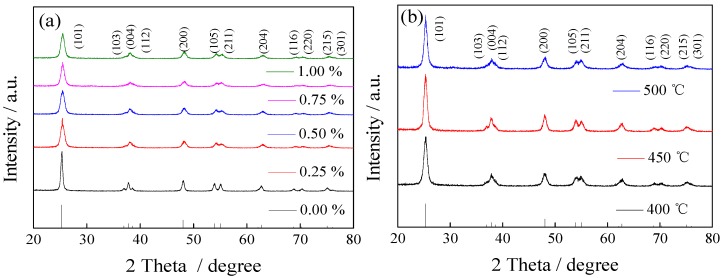
X-ray diffractometer (XRD) patterns of the samples: (**a**) La^3+^ with different dopant amounts calcined at 500 °C; (**b**) 0.75% La/TiO_2_ at different calcined temperatures.

The XRD patterns of different doping amounts of La (a) and different calcination temperatures (b) of La/TiO_2_ are shown in Figure 1. The major peaks at 2*θ* values of 25.21°, 36.75°, 37.53°, 38.40°, 47.87°, 53.53°, 54.86°, 62.37°, 68.30°, 70.03° and 74.63° corresponded to diffractions of the (101), (103), (004), (112), (200), (105), (211), (204), (116), (220), (215) and (301) planes of anatase TiO_2_, respectively (JCPDS card NO.21-1272) [27]. It is observed that the diffraction peak of pure TiO_2_ is relatively narrow, while it became broader and the relative intensity decreased with the increasing of the La doping amount, indicating a systematic decrease in grain sizes and the increase of specific surface area. The ionic radius of La^3+^ is 0.106 nm, which is larger than that of Ti^4+^ (which is 0.068 nm), so the doping ions would be difficult to get into the TiO_2_ lattice. The crystallite sizes of the samples were calculated by the Scherrer formula, Equation (2):

(2) $$D=\frac{k\lambda }{\beta \cos\theta }$$

where *D* is crystallite size; *k* is Scherrer constant, *k* = 0.89; *λ* is wavelength of X-ray; *β* is full width at half maximum of the peak (in radians); and *θ* is angle of diffraction (in degrees).

The crystallite size of pure TiO_2_ is 22 nm, and that of 0.75% La/TiO_2_ is 13 nm. As a result, the doping of La could inhibit the crystal growth of TiO_2_. Compared with pure TiO_2_, there is no new phase for the doped catalyst, indicating that La^3+^ might be in the form of small oxide clusters highly dispersed on the surface of TiO_2_, which would hinder the growth of TiO_2_ particles. The oxide also easily becomes light interception sub-trapping centers and ultimately affects the photocatalytic activity [28].

#### 3.1.2. XPS

**Figure 2:**
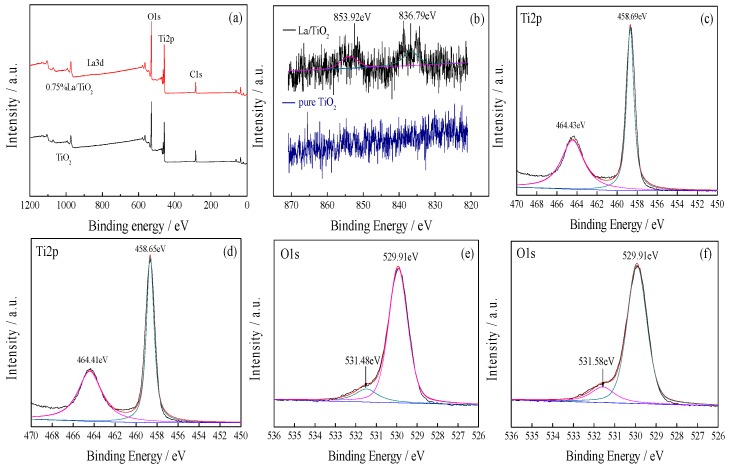
X-ray photoelectron spectroscopy (XPS): (**a**) survey spectra; (**b**) La3d; (**c**) Ti2p of pure TiO_2_; (**d**) Ti2p of La/TiO_2_; (**e**) O1s of pure TiO_2_; (**f**) O1s of La/TiO_2_.

The survey spectrum Figure 2a shows the predominant peaks corresponding to Ti, O and C. The strength of La is not obvious due to the low doping amount. A carbon element may come from the pollution of the X-ray photoelectron energy spectrum instrument. As seen from Figure 2b, after La doped, the main peaks at 853.92 and 836.79 eV are well in accordance with the standard XPS peaks of La^3+^. Figure 2c,d shows the Ti2p XPS spectra of pure TiO_2_ and 0.75% La/TiO_2_. The XPS spectrum of Ti2p for La/TiO_2_ could be fitted as two peaks that are composed by Ti2p1/2 and Ti2p3/2, which implied that almost all of the Ti atoms exist in the form of +4 valences. The peaks of pure TiO_2_ are at 464.43 eV and 458.69 eV, but the peaks are at 464.41 eV and 458.65 eV after doping. As seen from Figure 2e,f, different peaks appear after the fitting of O1s, which indicates that the samples of oxygen exist in different forms. The fitted strong peak of O1s located at 529.91 eV was the lattice oxygen in TiO_2_. The weak peak at 531.48 eV corresponded to the peak of adsorbed oxygen on the surface of TiO_2_. After doped, the peaks of the O1s spectrum shifted slightly to 529.92 eV and 531.58 eV, while the binding energy of Ti2p was smaller than that of pure TiO_2_. This might be due to the combination of La and Ti, which forms new chemical bonds of Ti-La, resulting in the electronic transfer from titanium atom to lanthanum atoms. The valence state of Ti^4+^ ions is slightly reduced when incorporated into the TiO_2_ lattice [29]. In addition, the doping of La combined with oxygen to form La_2_O_3_ on the surface of the sample. Since the ionic radius of La^3+^ is larger than that of Ti^4+^, it is difficult for La^3+^ to replace Ti^4+^ to form a stable solid solution [30]. However, Ti^4+^ could enter the lattice of La_2_O_3_, leading to the charge imbalance of the TiO_2_ lattice and production of Ti^3+^ [31]. It is well known that the adsorption of oxygen is very important for trapping the excited electron to suppress the recombination with hole. Thus, the doped catalyst has higher activity than the non-doped counterpart.

#### 3.1.3. UV-vis DRS

**Figure 3:**
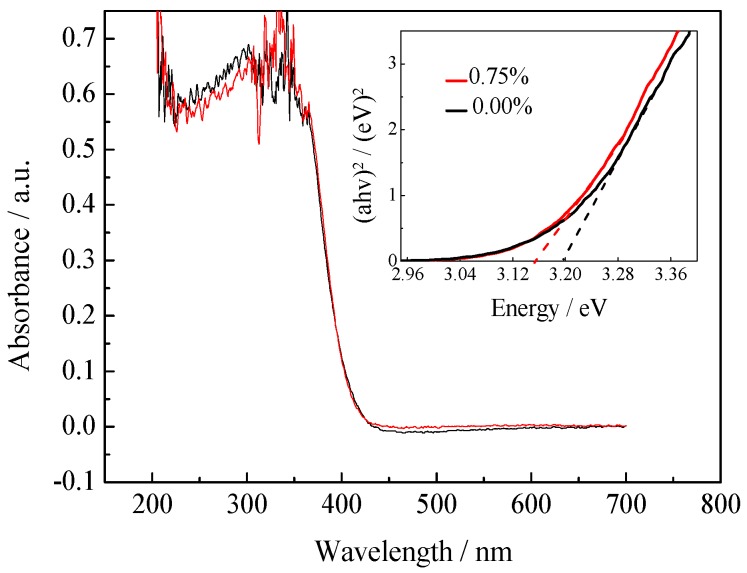
UV-vis diffuse reflectance spectroscopy (DRS) of pure TiO_2_ and 0.75% La/TiO_2_.

Figure 3 shows the UV-vis DRS of pure TiO_2_ and 0.75% La/TiO_2_ catalysts in the range of 200–700 nm. The data plots of absorption square versus energy in the absorption edge region are further estimated in the inset of Figure 3. The spectra of La/TiO_2_ indicate a little red shift in the band-gap transition compared with pure TiO_2_. A red shift of this type can be attributed to the charge-transfer transition between rare earth ions of 4f electrons and the TiO_2_ conduction or valence band. The light response range extends to the visible light, and the electron-hole pairs increased by the enhancement of light absorption ability. So, the photocatalytic activity might be improved [32,33]. The square of the absorption coefficient was linear with energy in the absorption edge region. Band-gap energies were deduced via extrapolating a straight line to the abscissa axis. The band-gap energy can be calculated by Equation (3) [34].

(3) $$ahv=A{\left(hv-Eg\right)}^{\frac{n}{2}}$$

where *a*, *h*, *v*, *A* and *E*g represent the absorption coefficient, Planck’s constant, light frequency, a constant, and band-gap energy, respectively. The value of n is determined by the type of optical transition of the semiconductor (*n* = 1) for direct transition, and *n* = 4 for indirect transition. The band-gap energies of pure TiO_2_ and 0.75% La/TiO_2_ were estimated to be 3.09 eV and 3.34 eV, respectively. This showed that the doping of La could narrow the band-gap of TiO_2_ and reduce the band-gap energy, which is important to slow down the recombination rate of the electron-hole pairs and ultimately enhance the photocatalytic activity [35].

#### 3.1.4. Specific Surface Area and Porosity Analysis

**Figure 4:**
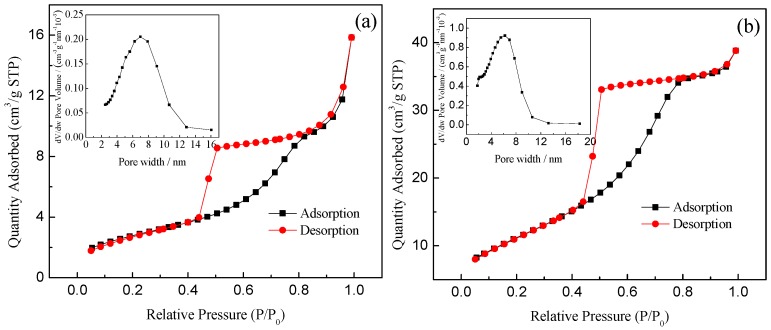
N_2_ adsorption-desorption isotherms and pore size distributions (inset) of pure TiO_2_ (**a**) and 0.75% La/TiO_2_ (**b**).

As seen from Figure 4, the N_2_ adsorption-desorption isotherms are characteristic of the typical Langmuir IV isotherm with hysteresis loop [36,37], which indicate that the synthesized samples have a mesoporous structure. The N_2_ adsorption-desorption isotherms of pure TiO_2_ and 0.75% La/TiO_2_ show the H2-type hysteresis loop with uniform particle accumulation in the hole. Generally speaking, it is considered as an inkbottle shaped channel with a small mouth and large cavity [38]. The BJH (Barrett-Joyner-Halenda) curve showed that the samples were with relatively narrow pore size distribution, and the mesoporous ranges from 2–10 nm. The most probable pore size of pure TiO_2_ was 6.99 nm, and the specific surface area was 10 m^2^·g^−1^. However, the most probable pore size of 0.75% La/TiO_2_ was 6.21 nm, and the specific surface area was 49 m^2^·g^−1^. The specific surface area of 0.75% La/TiO_2_ was significantly larger than that of pure TiO_2_. The relatively high surface area of La^3+^ doped samples confirmed that the frameworks of TiO_2_ have better adsorption ability. This may be due to the linkage between the rare earth ions and titanium by oxygen bridge, which effectively enhances the specific surface area of TiO_2_ [39]. The larger surface area, the more surface reaction sites, which is beneficial to improve the photocatalytic activity.

#### 3.1.5. Microstructure Analysis

**Figure 5:**
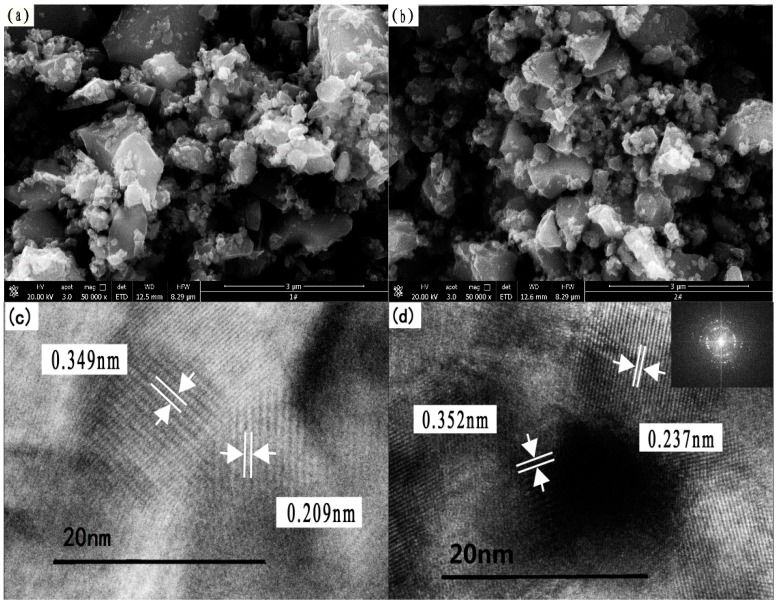
Scanning electron microscopy (SEM) and high-resolution transmission electron microscopy (HRTEM) images of samples: (**a**) SEM image of pure TiO_2_; (**b**) SEM image of 0.75% La/TiO_2_; (**c**) HRTEM micrograph of pure TiO_2_; (**d**) HRTEM micrograph of 0.75% La/TiO_2_.

Figure 5a,b shows the particulate morphology of pure TiO_2_ and the 0.75% La/TiO_2_. They display an irregular structure and contain a mixture of shaped particles [40]. The particle size of La/TiO_2_ was significantly smaller than that of pure TiO_2_, and the dispersion was better than that of pure TiO_2_. The doping of La may have decreased the particle size, increased the surface area and dispersion, which is in accordance with the results of XRD.

Figure 5c,d shows a high-resolution transmission electron microscopy (HRTEM) diagram of pure TiO_2_ and the 0.75% La/TiO_2_. In Figure 5c, the lattice fringe spacing of pure TiO_2_ is 0.349 nm and 0.209 nm, corresponding to the lattice planes (101) and (004) of anatase phase, respectively. In Figure 5d, the lattice fringe spacing of 0.75% La/TiO_2_ is mainly 0.352 nm and 0.237 nm. The average grain size of TiO_2_ was about 13–22 nm, which is consistent with the results of XRD analysis. The clear crystal lattice fringe suggests that the sample has good crystallinity. The fast Fourier transform (FFT) image in the inset of Figure 5d indicates that the sample is in a well-organized mesophase [41,42]. The FFT pattern also suggests the single crystal diffraction point, obviously. This suggests that the prepared mesostructure is a cubic phase oriented along the (101) and (004) directions, respectively [43,44].

### 3.2. Photocatalytic Degradation of Benzohydroxamic Acid

#### 3.2.1. Effect of La^3+^ Doping Amount

**Figure 6:**
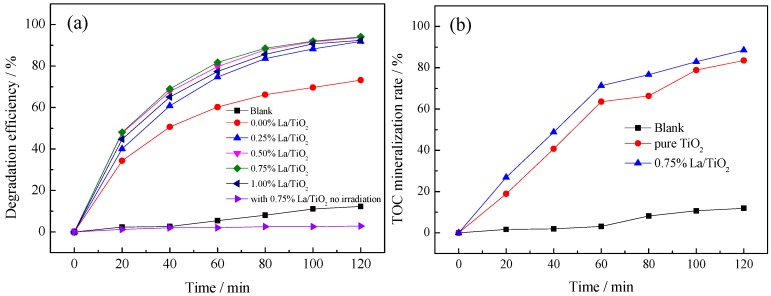
(**a**) Effect of different La doping ratio on the photodegradation of benzohydroxamic acid by La/TiO_2_; (**b**) TOC mineralization rates of 0.75% La/TiO_2_, pure TiO_2_ and blank. Reaction conditions: *C* (benzohydroxamic acid) = 30 mg·L^−1^, *C* (catalyst dosage) = 0.3 g·L^−1^, 300 W mercury lamp, calcined temperature at 500 °C.

The effects of different La^3+^ doping amounts on photocatalytic degradation of benzohydroxamic acid are as shown in Figure 6.

As observed in Figure 6a, the adsorption of benzohydroxamic acid on La/TiO_2_ is negligible. The photocatalytic activity of La/TiO_2_ is higher than that of pure TiO_2_. The degradation efficiency of the target pollutant first increased and then decreased as the doping amount of La from 0.00% to 1.00%, and 0.75% La/TiO_2_ indicated the highest photocatalytic activity. The increasing of doped La^3+^ amount would lead to an expansion of TiO_2_ lattice, which might produce crystal defects and distortion. Then, energy band structure would change and the recombination rate of electron-hole pairs would decrease. However, there would be too many defects due to the lattice distortion when excess La^3+^ was doped, and the photocatalytic activity would be inhibited. The degradation results give that the optimal doping ratio is 0.75%, which corresponded to the results of UV-vis DRS. In brief, the appropriate La doping amount not only avoids the waste of rare earth elements, but also improves the photocatalytic activity. In addition, the mineralization of benzohydroxamic acid was up to 88.5% by 0.75% La/TiO_2_, as depicted in Figure 6b. Zhou and Hu studied the biodegradation of benzonhydroxamic acid. They needed five or more days to degrade more than 85% of the pollutant under the conditions of additional nutritions [45,46]. So, the degradation of benzohydroxamic acid by photocatalytic oxidation with TiO_2_-based catalysts has a significant advantage.

#### 3.2.2. Effect of Light Intensity

**Figure 7:**
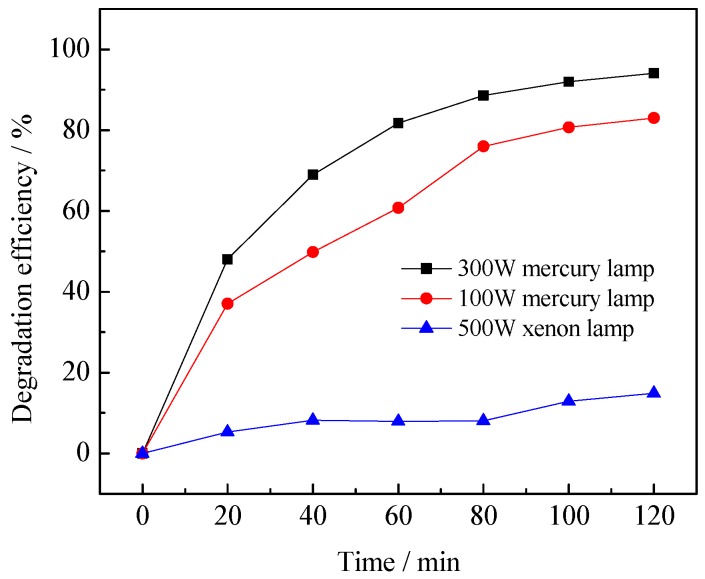
Effect of light intensity on photocatalytic degradation of benzohydroxamic acid. Reaction conditions: *C* (benzohydroxamic acid) = 30 mg·L^−1^, *C* (catalyst dosage) = 0.3 g·L^−1^, 0.75% La/TiO_2_, calcined temperature at 500 °C.

The effects of different light intensity on photocatalytic degradation of benzohydroxamic acid are as shown in Figure 7.

As can be seen from Figure 7, the degradation rate of benzohydroxamic acid under the irradiation of a mercury lamp is better than under a xenon lamp. The light intensity of the 300 W mercury lamp and the 100 W mercury lamp were 5.6 mW·cm^−2^ and 0.46 mW·cm^−2^, respectively, while the light intensity of the 500 W xenon lamp was 39.5 mW·cm^−2^. The wavelength distribution of the mercury lamps and the xenon lamp is listed in Table S1 and Figure S2 respectively in the Supplementary Material. The photocatalytic activity increased with the increase of light intensity under the same light source. Because the light-response range of La/TiO_2_ is mainly in the ultraviolet or near ultraviolet band, the number of photons increased with the increase of light intensity, and holes increased accordingly. This produced much more ·OH. So the degradation rate of benzohydroxamic acid increased.

#### 3.2.3. Effect of Catalyst Dosage

**Figure 8:**
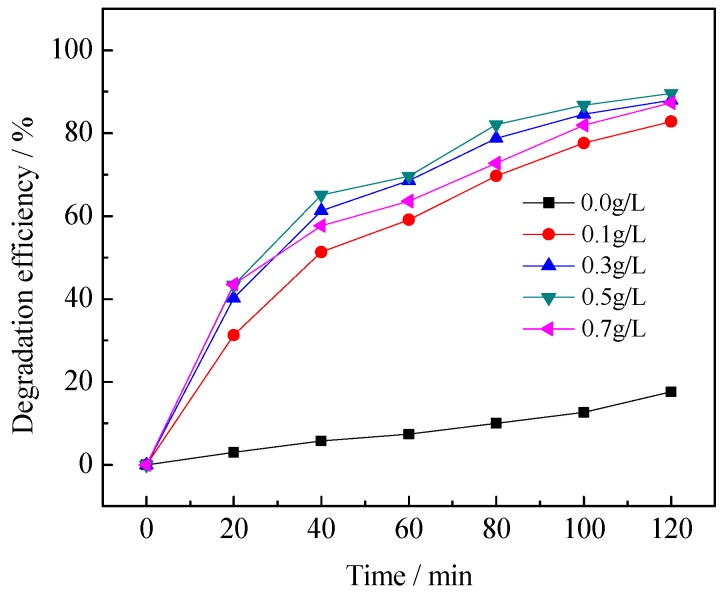
Effect of La/TiO_2_ dosage on photocatalytic degradation of benzohydroxamic acid. Reaction conditions: *C* (benzohydroxamic acid) = 30 mg·L^−1^, 300 W mercury lamp, 0.75% La/TiO_2_, calcined temperature at 500 °C.

Figure 8 shows the variations in the ratio of degradation at different dosages of photocatalyst ranging from 0 to 0.7 g·L^−1^. It can be seen that the photocatalytic degradation efficiency increases with an increasing amount of the 0.75% La/TiO_2_ photocatalyst and reaches the highest value when the concentration is 0.5 g·L^−1^. There are three reasons to explain this: (1) the smaller dosage of La/TiO_2_ generates less electron-hole pairs, which leads to the lower photocatalytic activity; (2) the availability of active sites increase with the increase of La/TiO_2_ dosage; (3) overload of the photocatalysts would decrease the light penetration and increase radiation scattering by the suspension catalyst and finally reduce the degradation rate.

#### 3.2.4. Effect of Initial pH Value of Solution

**Figure 9:**
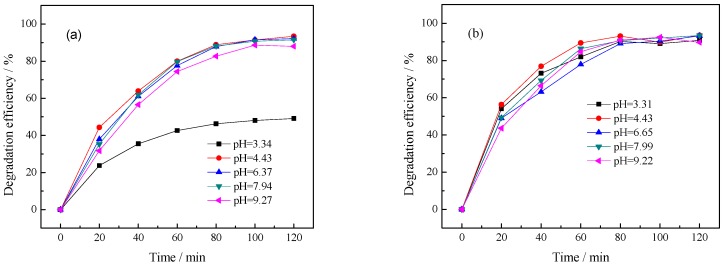
Effect of initial pH value on photocatalytic degradation of benzohydroxamic acid: (**a**) the pH value was adjusted by HNO_3_ and NaOH; (**b**) the pH value was adjusted by HCl and NaOH). Reaction conditions: *C* (benzohydroxamic acid) = 30 mg·L^−1^, *C* (catalyst dosage) = 0.5 g·L^−1^, 300 W mercury lamp, 0.75% La/TiO_2_, calcined temperature at 500 °C.

As can be seen from Figure 9, the pH values have different effects on the degradation of benzohydroxamic acid when the pH value was adjusted by a different regulator. In general, the highest photocatalytic activity can be reached when the pH of benzohydroxamic acid solution is 4.43 (original pH value of 30 mg·L^−1^ benzohydroxamic acid solution). The basic solution adjusted by NaOH almost shows no effect on the degradation of benzohydroxamic acid. However, there exists an interesting phenomenon: the degradation of benzohydroxamic acid was suppressed at stronger acidic conditions with HNO_3_ as the regulator (Figure 9a), while there was almost no change with HCl as the regulator (Figure 9b). In order to clarify the difference, another experiment using two anions including Cl^−^ and NO_3_^−^ in their sodium salt form was designed to investigate different effects on the photodegradaton of benzohydroxamic acid by La/TiO_2_ (the relative figure is listed as Figure S3 in the Supplementary Materials). As the result, NO_3_^−^ indicates obviously inhibitory effect compared with Cl^−^. As seen from the structure of benzohydroxamic acid (Figure S1), there exist N-containing functional groups in the molecular. NO_3_^−^, added from HNO_3_ or NaNO_3_, would compete the adsorptive sites on the surface of La/TiO_2_ with the N-containing functional groups from the target pollutant.

#### 3.2.5. The Reusability of Photocatalyst

**Figure 10:**
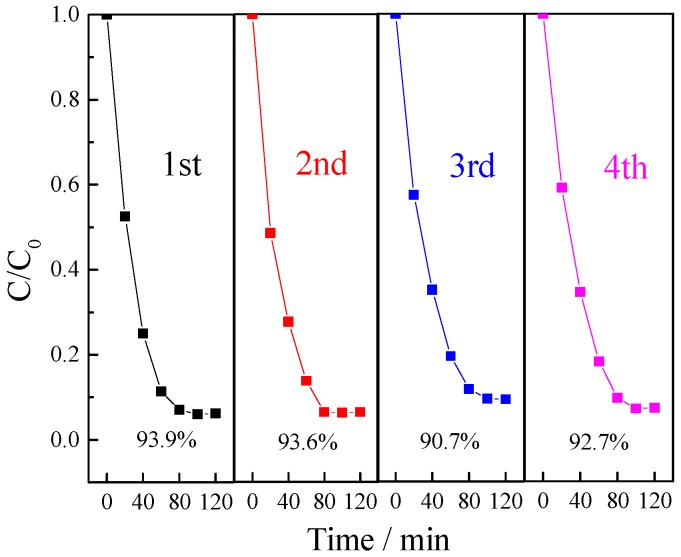
Recycle runs in the photocatalytic degradation of benzohydroxamic acid by La/TiO_2_. Reaction conditions: *C* (benzohydroxamic acid) = 30 mg·L^−1^, *C* (catalyst dosage) = 0.5 g·L^−1^, 300 W mercury lamp, 0.75% La/TiO_2_, calcined temperature at 500 °C.

Except for the activity of photocatalysts, the reusability is meaningful to investigate for their practical application. Therefore, four successive recycling tests for the degradation of benzohydroxamic acid by La/TiO_2_ were performed. As shown in Figure 10, the removal efficiencies were 93.9%, 93.6%, 90.7%, and 92.7% for the first to the fourth runs, respectively. The degradation efficiency decreased about 3.2% after four cycles. A gradually decreasing trend can be found from the results of degradation efficiency, but the differences among the fourth runs were not obvious, which indicates that La/TiO_2_ possesses a good stability and reusability in the photodegradation of benzohydroxamic acid.

#### 3.2.6. Fourier Transform Infrared Spectroscopy (FTIR) Analysis and Interfacial Charge Transfer Processes

**Figure 11:**
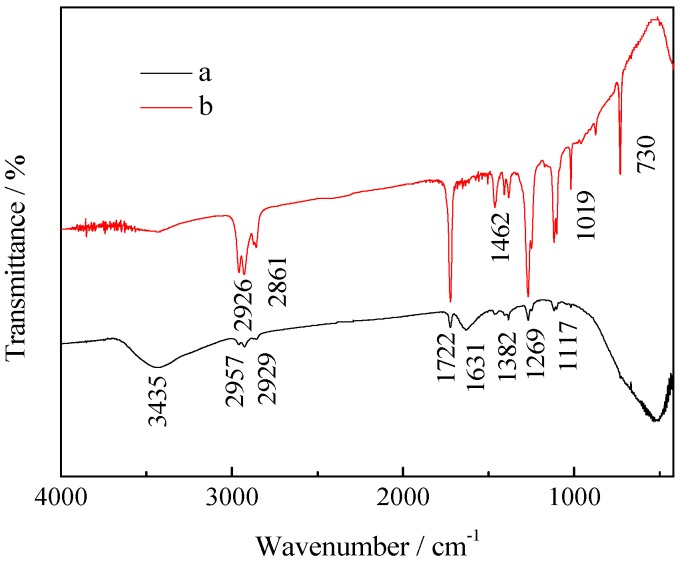
Fourier Transform Infrared Spectroscopy (FTIR) spectra of samples: (**a**) 0.75% La/TiO_2_; (**b**) photocatalytic degradation of benzohydroxamic acid by 0.75% La/TiO_2_ after 2 h.

Figure 11 shows the FTIR spectra of 0.75% La/TiO_2_ (Curve a) and after 2 hours’ photocatalytic degradation of benzohydroxamic acid by 0.75% La/TiO_2_ (Curve b). The following information could be given by the analysis of FTIR spectra [47,48]: the characteristic peak of TiO_2_ is 400–800 cm^−1^, caused by the stretching vibration and bending vibration of Ti-O-Ti and Ti-O bond; the absorption located at 3435 cm^−1^ characterizes the hydroxyl groups of Ti-OH at weak surface active sites, with physisorbed water molecules bound by weak hydrogen bonds with OH^–^ groups of TiO_2_ surfaces; the characteristic peaks of 1117, 1269 and 1722 cm^−1^ corresponding to the Ti-O-C, the C-O, and C=O in Curve b are stronger than those in Curve a; 730 cm^−1^ may be a long carbon chain of CH_2_ in Curve b; stretching vibration of CH_3_ groups is around 2926 cm^−1^ and 2961 cm^−1^. All the information above could suggest that the benzene ring in benzohydroxamic acid had been fractured, and some alkanes or ester compounds had been generated by the photocatalytic reaction.

**Figure 12:**
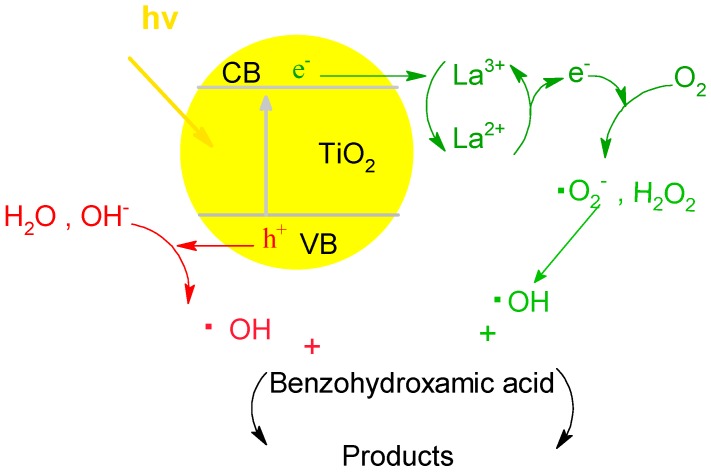
Schematic representation of the interfacial charge transfer processes in the La/TiO_2_.

Figure 12 illustrates the possible interfacial charge transfer processes. Element La exists in the stable form of La^3+^ as described in the section of XRD and XPS, which might trap the photoexcited electrons to produce La^2+^. However, La^2+^ is relatively unstable: the electrons can be easily detrapped and transfer to O_2_ adsorbed on the surface of TiO_2_ to produce the ·O_2_^−^ [49]. ·O_2_^−^ and the photoexcited holes would react with H_2_O, OH^−^, or H_2_O_2_ to produce ·OH. Then, benzohydroxamic acid would be oxided to products including CO_2_ and H_2_O. The doped La^3+^ could increase the electron transfer from the surface of the catalyst and decrease the recombination of photogenerated electrons and holes.

## 4. Conclusions

La-doped TiO_2_ photocatalysts were synthesized by the sol-gel method. XRD diffraction peaks of doped TiO_2_ were broader and the relative intensity was weaker than pure TiO_2_. It might improve the thermal stability of the anatase phase of TiO_2_, suppress particle aggregation, grain growth of TiO_2_ and increase the specific area of TiO_2_. The red shift of La/TiO_2_ in the band-gap transition could reduce gap energy of TiO_2_ and improve the response strength and threshold value of TiO_2_ for visible light and extend its absorption side band. La/TiO_2_ also had a larger specific surface area and a more regular shape in morphology. Furthermore, 0.75% La/TiO_2_ (500 °C) indicated the highest photocatalytic degradation ability to benzohydroxamic acid: the removal rate and mineralization efficiency of benzohydroxamic acid reached 94.1% and 88.5%, respectively, at the conditions of pH 4.43, 30 mg·L^−1^ of benzohydroxamic acid, 0.5 g·L ^−1^ of catalyst, and the irradiation of 300 W mercury lamp. The doping of La^3+^ could reduce the recombination of photoexited electrons and holes, resulting in the improvement of removal efficiency of benzohydroxamic acid by La/TiO_2_.

## Supplementary Materials

The following are available online at www.mdpi.com/1660-4601/13/10/997/s1, Table S1: The emission range of 100 W and 300 W Mercury, Figure S1: Structure of benzohydroxamic acid, Figure S2: The emission range of the 500 W xenon lamp, Figure S3: Effect of inorganic anions Cl^−^ and NO_3_^−^ on photocatalytic degradation of benzohydroxamic acid.

## References

[1] Assis S.M., Montennegro L.C.M., Peres A.E.C. (1996). Utilization of hydroxamates in minerals froth flotation. Miner. Eng..

[2] Quast K.B. (2000). A review of hematite flotation using 12-carbon chain collectors. Miner. Eng..

[3] Hashimoto S., Nakumura Y. (1995). Nuclease activity of a hydroxamic acid derivative in the presence of various metal ions. Chem. Soc. Chem. Commun..

[4] Lee K., Archibald D., McLean J. (2009). Flotation of mixed copper oxide and sulphidemineral swith xanthate and hydroxamate collectors. Miner. Eng..

[5] Liu X., Zhang Y., Li J., Zhao J., Xi N., He D. (2015). Synthesis and crystal structure of *N*-((3-(2-nitrophenyl) propanoyl)oxy)-*N*-phenylbenzamide. Chin. J. Struct. Chem..

[6] Sinirtas E., Isleyen M., Soylu G.S.P. (2016). Photocatalytic degradation of 2,4-dichlorophenol with V_2_O_5_-TiO_2_ catalysts: Effect of catalyst support and surfactant additives. Chin. J. Catal..

[7] Ako R.T., Ekanayake P., Tan A.L. (2016). La modified TiO_2_ photoanode and its effect on DSSC performance: A comparative study of doping and surface treatment on deep and surface charge trapping. Mater. Chem. Phys..

[8] Abdullah H., Khan M.R., Pudukudy M., Yaakob Z., Ismail N.A. (2015). CeO_2_-TiO_2_ as a visible light active catalyst for the photoreduction of CO_2_ to methanol. J. Rare Earths.

[9] Salas-Bañales E., Quiroz-Segoviano R.I.Y., Díaz-Alejo L.A., Rojas-González F., Estrella-González A., Campero A., García-Sánchez M.A. (2015). Comparative study of the optical and textural properties of tetrapyrrole macrocycles trapped within ZrO_2_, TiO_2_, and SiO_2_ translucent xerogels. Molecules.

[10] Golubovic A., Tomic N., Fincur N. (2014). Synthesis of pure and La-doped anatase nanopowders by sol-gel and hydrothermal methods and their efficiency in photocatalytic degradation of alprazolam. Ceram. Int..

[11] Huo Y., Zhu J., Li J. (2007). An active La/TiO_2_ photocatalyst prepared by ultrasonication-assisted sol-gel method followed by treatment under supercritical conditions. J. Appl. Phys..

[12] Okuno T., Kawamura G., Muto H., Matsuda A. (2014). Fabrication of shape-controlled Au nanoparticles in a TiO_2_-containing mesoporous template using UV irradiation and their shape-dependent photocatalysis. J. Mater. Sci. Technol..

[13] Li H., Shi Z., Liu H. (2010). Humidity sensing properties of La^3+^/Ce^3+^-doped TiO_2_-20 wt.% SnO_2_ thin films derived from sol-gel method. J. Rare Earths.

[14] Bingham S., Daoud W.A. (2011). Recent advances in making nano-sized TiO_2_ visible-light active through rare-earth metal doping. J. Mater. Chem..

[15] Stengl V., Bakardjieva S., Murafa N. (2008). Preparation and photocatalytic activity of rare earth doped TiO_2_ nanoparticles. Mater. Chem. Phys..

[16] El-Bahy Z.M., Ismail A.A., Mohamed R.M. (2008). Enhancement of titania by doping rare earth for photodegradation of organic dye (Direct Blue). J. Hazard. Mater..

[17] Du P., Bueno-Lopez A., Verbaas M. (2008). The effect of surface OH-population on the photocatalytic activity of rare earth-doped P25-TiO_2_ in methylene blue degradation. J. Catal..

[18] Zhang J., Zhao Z., Wang X., Yu T., Guan J., Yu Z., Li Z., Zou Z. (2010). Increasing the oxygen vacancy density on the TiO_2_ surface by La-doping for dye-sensitized solar cells. J. Phys. Chem..

[19] Grujic-Brojcin M., Armakovic S., Tomic N. (2013). Surface modification of sol-gel synthesized TiO_2_ nanoparticles induced by La-doping. Mater. Charact..

[20] Li S., Xu Y., Wang X. (2016). Catalytic degradation of 4-chlorophenol with La/TiO_2_ in a dielectric barrier discharge system. RSC Adv..

[21] Priyanka K.P., Revathy V., Rosmin V. (2016). Influence of La doping on structural and optical properties of TiO_2_ nanocrystals. Mater. Charact..

[22] Leila E., Hinda L., Ammar H. (2015). Synthesis, characterization and photocatalytic activity of Li^−^, Cd^−^, and La-doped TiO_2_. Mater. Sci. Semicond. Process..

[23] Moradi S., Vossoughi M., Feilizadeh M. (2015). Photocatalytic degradation of dibenzothiophene using La/PEG-modified TiO_2_ under visible light irradiation. Res. Chem. Intermed..

[24] Kumaresan L., Prabhu A., Palanichamy M., Arumugam E. (2010). Synthesis and characterization of Zr^4+^, La^3+^ and Ce^3+^ doped mesoporous TiO_2_: Evaluation of their photocatalytic activity. J. Hazard. Mater..

[25] Khalid N.R., Ahmed E., Hong Z.L., Ahmad M. (2012). Synthesis and photocatalytic properties of visible light responsive La/TiO_2_-graphene composites. Appl. Surf. Sci..

[26] Tanyi A.R., Rafieh A.I., Ekaneyaka P. (2015). Enhanced efficiency of dye-sensitized solar cells based on Mg and La co-doped TiO_2_ photoanodes. Electrochim. Acta.

[27] Fan W., Bai H., Zhang G., Yan Y., Liu C., Shi W. (2014). Titanium dioxide macroporous materials doped with iron: Synthesis and photo-catalytic properties. Cryst. Eng. Comm..

[28] Sharotri N., Sud D. (2015). A greener approach to synthesize visible light responsive nanoporous S-doped TiO_2_ with enhanced photocatalytic activity. New J. Chem..

[29] Dhanasekaran P., Selvaganesh S.V., Giridhar V.V., Bhat S.D. (2016). Iron and nitrogen co-doped titania matrix supported Pt for enhanced oxygen reduction activity in polymer electrolyte fuel cells. RSC Adv..

[30] Komarala E.V.P., Doshi S., Mohammed A., Bahadur D. (2016). Efficient antibacterial activity via protein degradation of a 3D layered double hydroxide-reduced graphene oxide nanohybrid. RSC Adv..

[31] Guerra V.L.P., Altamura D., Trifiletti V. (2015). Implications of TiO_2_ surface functionalization on polycrystalline mixed halide perovskite films and photovoltaic devices. J. Mater. Chem..

[32] Feng Y., Liu C., Chen J., Che H., Xiao L., Gu W., Shi W. (2016). Facile synthesis of BiOI/CdWO_4_ p-n junctions: Enhanced photocatalytic activities and photoelectrochemistry. RSC Adv..

[33] Wen Q., Yu J., Sun X. (2016). Hydrothermal treatment of a TiO_2_ film by hydrochloric acid for efficient dye-sensitized solar cells. New J. Chem..

[34] Spadavecchia F., Cappelletti C., Ardizzone S. (2010). Solar photoactivity of nano-N-TiO_2_ from tertiary amine: Role of defects and paramagnetic species. Appl. Catal. B.

[35] Gomez V., Clemente A., Irusta S., Balasab F., Santamaria J. (2014). Identification of TiO_2_ nanoparticles using La and Ce as labels: Application to the evaluation of surface contamination during the handling of nanosized matter. Environ. Sci. Nano.

[36] Das S.K., Bhunia M.K., Sinha A.K., Bhaumik A. (2011). Characterization, and biofuel application of mesoporous Zirconium oxophosphates. ACS Catal..

[37] Masika E., Mokaya R. (2011). Mesoporous aluminosilicates from a zeolite BEA recipe. Chem. Mater..

[38] Li X., Lin H., Chen X., Niu H., Zhang T., Liu J., Qu F. (2015). Fabrication of TiO_2_/porous carbon nanofibers with superior visible photocatalytic activity. New J. Chem..

[39] Bai S., Hu X., Sun J., Ren B. (2014). Preparation and characterization of Ti supported bimodal mesoporous catalysts using a self-assembly route combined with a ship-in-a-bottle method. New J. Chem..

[40] Zhou X., Wang G., Guo L. (2014). Hierarchically structured TiO_2_ for Ba-filled skutterudite with enhanced thermoelectric performance. J. Mater. Chem. A.

[41] Hasan M.R., Hamid S.B.A., Basirun W.J. (2015). Ga doped RGO-TiO_2_ composite on an ITO surface electrode for investigation of photoelectrocatalytic activity under visible light irradiation. New J. Chem..

[42] Li N., Jayaraman S., Tee S.Y. (2014). Effect of La-doping on optical bandgap and photoelectrochemical performance of hematite nanostructures. J. Mater. Chem. A.

[43] Boulon G., Alombert-Goget G., Guyot Y., Guzik M., Epicier T., Blanchard N.P., Chen L., Hud V., Chen W. (2014). Conjugation of TEM-EDX and optical spectroscopy tools for the localization of Yb^3+^, Er^3+^ and Co^2+^ dopants in laser glass ceramics composed of MgAl_2_O_4_ spinel nano-crystals embedded in SiO_2_ glass. J. Mater. Chem. C.

[44] Yoon S., Manthiram A. (2011). Hollow core-shell mesoporous TiO_2_ spheres for lithium ion storage. J. Phys. Chem. C.

[45] Zhou W. (2012). Study on the Biodegradability of Hydroxamic Acid Collectors. Ph.D. Thesis.

[46] Hu C., Wang C., Gong W., Mei G. (2013). Study on the biodegradation of three hydroxamic acid collectors. Hubei Agric. Sci..

[47] Li G., Li L., Boerio-Goates J., Woodfield B. (2005). High purity anatase TiO_2_ nanocrystals:  Near room-temperature synthesis, grain growth kinetics, and surface hydration chemistry. J. Am. Chem. Soc..

[48] Beaussart A., Petrone L., Mierczynska-Vasilev A., McQuillan A., David A. (2012). In situ ATR FTIR study of dextrin adsorption on anatase TiO_2_. Langmuir.

[49] Chen X., Liu Z., Tang J., Teng C., Cai T., Deng Q. (2015). La-modified mesoporous TiO_2_ nanoparticles with enhanced photocatalytic activity for elimination of VOCs. J. Porous Mater..

