# Supplementary material for: Degradation and Mineralization of Benzohydroxamic Acid by Synthesized Mesoporous La/TiO2
Source: Int J Environ Res Public Health. 2016 Oct 10;13(10):997. doi: 10.3390/ijerph13100997 (PMC5086736; doi:10.3390/ijerph13100997)
Supplement: Supplementary file 1 [file ijerph-13-00997-s001.pdf]

Supplementary Materials: Degradation and Mineralization of Benzohydroxamic Acid by Synthesized Mesoporous La/TiO_2

Xianping Luo, Junyu Wang, Chunying Wang, Sipin Zhu, Zhihui Li, Xuekun Tang and Min Wu

Table S1. The emission range of 100 W and 300 W Mercury.

Intensity (%)	Wavelength (nm)
15.3	265.2–265.5
16.6	296.7
23.9	302.2–302.8
49.9	312.6–313.2
100.0	365.0–366.3
42.2	404.5–407.8
77.5	435.8
546.1	577.0–579.0
93.0	76.5

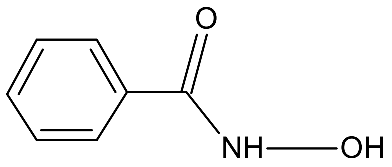

Figure S1. Structure of benzohydroxamic acid.

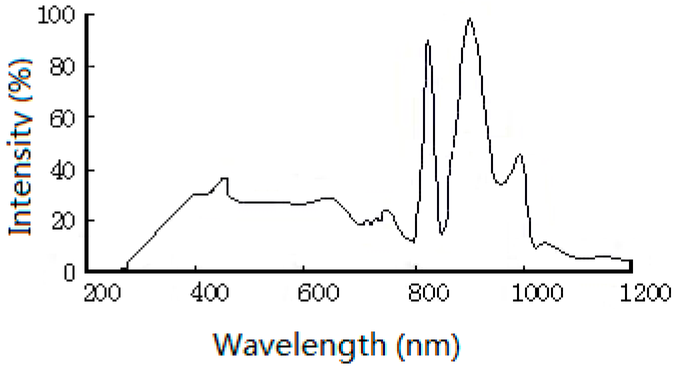

Figure S2. The emission range of the 500 W xenon lamp.

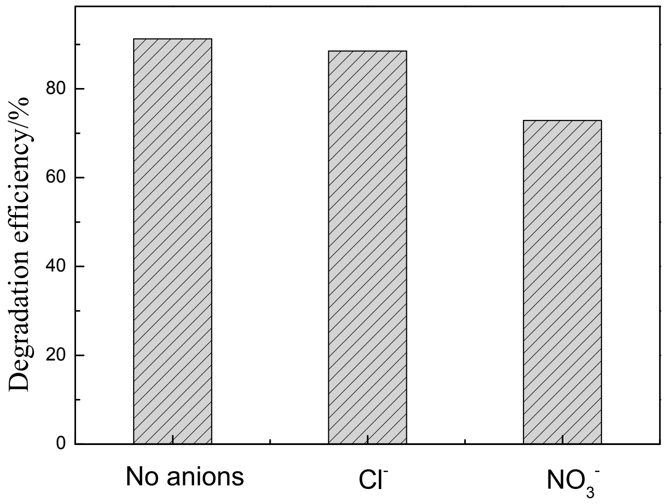

Figure S3. Effect of inorganic anions Cl^- and NO_3^- on photocatalytic degradation of benzohydroxamic acid.

© 2016 by the authors; licensee MDPI, Basel, Switzerland. This article is an open access article distributed under the terms and conditions of the Creative Commons by Attribution (CC-BY) license (http://creativecommons.org/licenses/by/4.0/)
